# Optical control of neuronal excitation and inhibition using a single opsin protein, ChR2

**DOI:** 10.1038/srep03110

**Published:** 2013-10-31

**Authors:** Holly Liske, Xiang Qian, Polina Anikeeva, Karl Deisseroth, Scott Delp

**Affiliations:** 1 Department of Mechanical Engineering, Stanford University, Stanford, California 94305; 2 Department of Anesthesiology, Perioperative and Pain Medicine, Stanford University, Stanford, California 94305; 3 Department of Materials Science and Engineering, Massachusetts Institute of Technology, Cambridge, Massachusetts 02139; 4 Department of Psychiatry and Behavioral Sciences, Stanford University, Stanford, California 94305; 5 Department of Bioengineering, Stanford University, Stanford, California 94305

## Abstract

The effect of electrical stimulation on neuronal membrane potential is frequency dependent. Low frequency electrical stimulation can evoke action potentials, whereas high frequency stimulation can inhibit action potential transmission. Optical stimulation of channelrhodopsin-2 (ChR2) expressed in neuronal membranes can also excite action potentials. However, it is unknown whether optical stimulation of ChR2-expressing neurons produces a transition from excitation to inhibition with increasing light pulse frequencies. Here we report optical inhibition of motor neuron and muscle activity *in vivo* in the cooled sciatic nerves of *Thy1-ChR2-EYFP* mice. We also demonstrate all-optical single-wavelength control of neuronal excitation and inhibition without co-expression of inhibitory and excitatory opsins. This all-optical system is free from stimulation-induced electrical artifacts and thus provides a new approach to investigate mechanisms of high frequency inhibition in neuronal circuits *in vivo* and *in vitro*.

The ability to both excite and inhibit neuronal activity with electrical stimulation of different frequencies has important implications for basic and clinical neuroscience. Low frequency electrical stimulation depolarizes neuronal membranes and evokes action potentials, whereas higher frequencies, ranging from 200 Hz to 30 kHz^1^ as well as continuous depolarizing stimulation, have been used to inhibit action potentials in both central^2^,^3^ and peripheral neurons^1^,^4^,^5^,^6^,^7^,^8^,^9^,^10^,^11^,^12^,^13^,^14^,^15^,^16^,^17^. High frequency electrical stimulation has been clinically implemented^18^,^19^,^20^ for cases in which inhibition of neuronal activity is desired, such as the treatment of pain^19^,^20^. While this is a valuable approach, the mechanisms underlying the frequency-dependent transition from excitation to inhibition remain unclear, in part due to stimulation-induced electrical artifacts that interfere with electrophysiological recordings^2^,^12^,^21^. Furthermore, low and high frequency electrical stimulation have limited ability to control activity in defined subsets of collocated neurons. Optogenetics is a powerful technique enabling activation and inhibition of specific cell types that are collocated^22^,^23^.

Previous studies have sought to simultaneously excite and inhibit a population of neurons using optogenetics^24^,^25^,^26^,^27^. These efforts used specialized viral and genetic approaches to achieve co-expression of ChR2 with an inhibitory opsin, such as halorhodopsin (NpHR), in the same neurons. Thus far, co-expression has been achieved *in vitro*^24^,^25^,^26^ and *in vivo* in zebrafish^27^. Excitation and inhibition were achieved in these studies by optical stimulation of two opsins with two light wavelengths of sufficient spectral separation. While optical stimulation of ChR2 is typically used to evoke neuronal activity, several studies observed inhibition of neuronal activity during high frequency blue light stimulation of ChR2-expressing neurons in the mouse brain^28^,^29^,^30^. In most cases the inhibition was produced by excitation of interneurons (e.g. cholinergic or parvalbumin neurons) that have inhibitory effects on the population activity or by excitation of broad neuronal populations that include inhibitory cells. To date there has been no systematic analysis to determine if the light pulse frequency of optical stimulation could be manipulated to achieve both excitation and inhibition using ChR2.

We sought to determine if optical stimulation of ChR2-expressing neurons exhibits a transition from neuronal excitation to inhibition with increasing light pulse frequency. We examined this in the mouse sciatic nerve, a system previously used to demonstrate orderly recruitment of motor units by blue light stimulation of ChR2^31^ and inhibition of motor neurons by green light stimulation of an enhanced NpHR^32^. Importantly, there are no inhibitory neurons in the sciatic nerve that could be responsible for inhibition. We first addressed whether blue light stimulation of ChR2-expressing neurons in transgenic *Thy1-ChR2-EYFP* mice could inhibit electrically evoked motor neuron and muscle activity. We next examined inhibition using a range of light pulse frequencies and continuous light and examined the role of nerve temperature in achieving inhibition. Lastly, we demonstrated simultaneous, all-optical control of neuronal excitation and inhibition *in vivo* in a *Thy1-ChR2-EYFP* mouse using 1 Hz pulses of blue light at the proximal nerve to evoke action potentials and a continuous 5 s blue light pulse at the distal nerve to inhibit action potential transmission and muscle activity. Our system capitalizes on the spatial and temporal precision of optogenetics^23^,^33^ while achieving excitation and inhibition using a single opsin and light wavelength.

## Results

### Optical inhibition using ChR2

**Figure 1:**
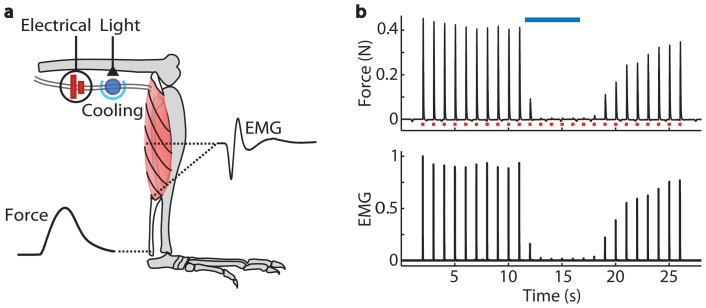
Optical inhibition of electrically evoked motor neuron and muscle activity in *Thy1-ChR2-EYFP* mice with blue light. (a) Schematic of experimental measures during electrical excitation and optical inhibition in a *Thy1-ChR2-EYFP* mouse. Motor axons were electrically stimulated with a stimulation cuff around the proximal sciatic nerve of an anesthetized mouse. The distal nerve was cooled and illuminated with blue laser light (465 nm). Fine wire EMG electrodes recorded electrical activity of the medial gastrocnemius. A force transducer attached at the Achilles tendon recorded contractile force of the lower limb muscles. (b) Example force and EMG data recorded during muscle twitches elicited by 25 s of 1 Hz, maximal electrical stimulation (0.1 ms pulse duration, 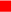, n = 1 mouse, 1 trial). EMG was full-wave rectified, filtered, and normalized to the maximum amplitude recorded during the trial. Average force and EMG were inhibited to 1% and 2%, respectively, of the pre-light amplitudes during 5 s of 50 Hz pulses of blue light (2 ms pulse duration, 7 mW/mm^2^, 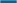) with the nerve cooled to 7°C.

In our preliminary investigations, we observed unreliable inhibition of electrically evoked muscle twitch force and electromyographic (EMG) activity during high frequency light pulses or continuous light in *Thy1-ChR2-EYFP* mice. While we observed inhibition in some trials, optical stimulation in other trials enhanced the electrically evoked twitch force and EMG. We identified that precise control of nerve temperature was important for achieving reliable optical inhibition. With the nerve cooled, we observed reliable optical inhibition of electrically evoked twitch force and EMG by illuminating the nerve with blue light (Fig. 1a). Twitch force and EMG amplitudes were inhibited during 5 s of 50 Hz light pulses (2 ms pulse duration, 7 mW/mm^2^) with the nerve maintained at 7°C throughout the trial. Example traces show inhibition of twitch force and EMG amplitudes to 1% and 2%, respectively, of pre-light amplitudes (Fig. 1b). Thus, 50 Hz optical stimulation of motor neurons expressing ChR2 with blue light is capable of inhibiting electrically evoked motor neuron and muscle activity.

### Effect of light pulse frequency on optical inhibition

**Figure 2:**
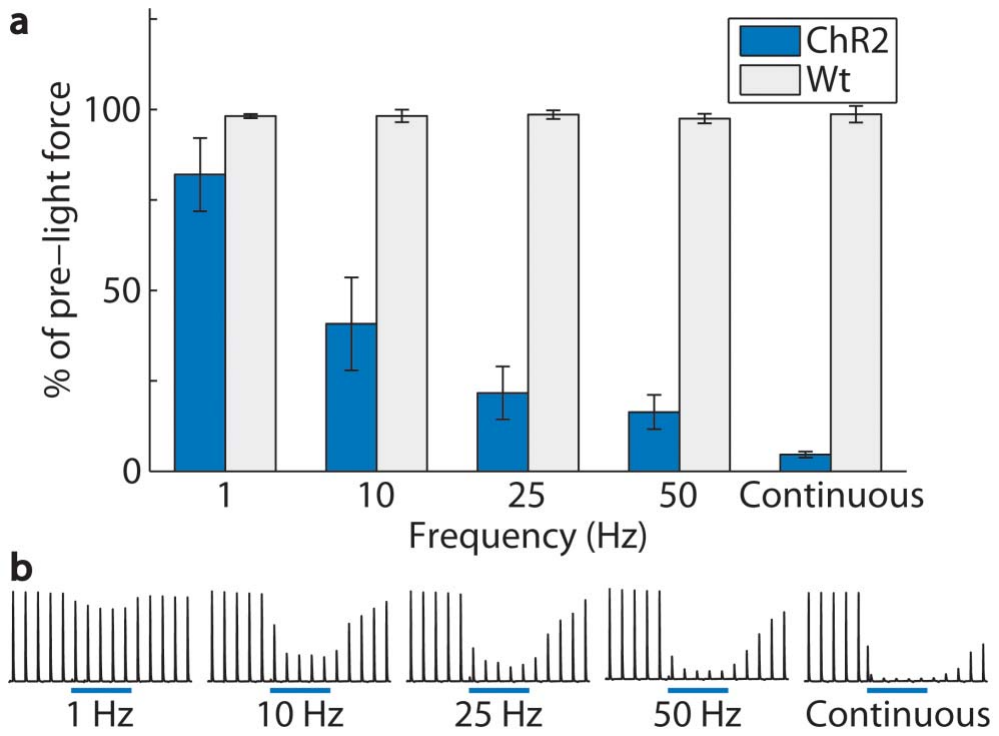
Inhibition increased with increasing light pulse frequency from 1 to 50 Hz (2 ms pulse duration, 7 mW/mm^2^) in *Thy1-ChR2-EYFP* mice (n = 6 mice, 90 trials). Inhibition was greatest during continuous light (5 s pulse duration). (a) At 1, 10, 25, and 50 Hz and continuous light, average twitch force amplitudes were inhibited to 82 ± 10, 41 ± 13, 22 ± 7, 16 ± 5, and 5 ± 1% of the pre-light amplitudes. Average twitch force amplitudes in wild-type mice (Wt, n = 4 mice, 60 trials) were unchanged during optical stimulation (98 ± 1, 98 ± 2, 99 ± 1, 97 ± 1, and 99 ± 2% at 1, 10, 25, and 50 Hz and continuous light). Pre-light twitch force amplitudes were approximately maximal in all trials, and nerve temperature was approximately 10°C (10 ± 2°C in ChR2 mice and 11 ± 1°C in wild-type mice). Error bars represent mean ± SEM calculated from the within mouse variances of mean. (b) Example traces from one mouse show average force amplitudes of 83, 29, 19, 9, and 3% of pre-light amplitudes at 1, 10, 25, and 50 Hz and continuous light.

**Figure 3:**
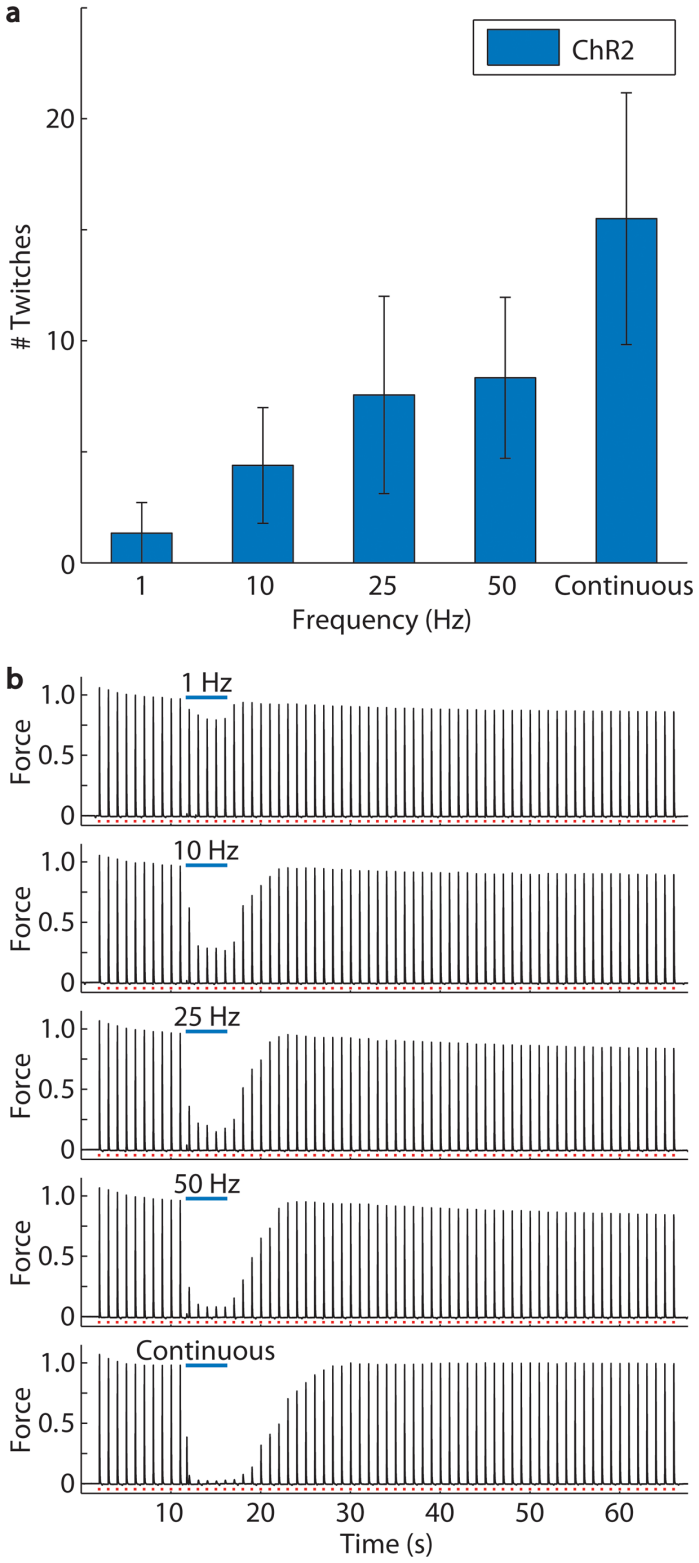
A greater number of post-light twitches was required for recovery of force amplitude after optical inhibition using increasing light pulse frequencies while the nerve was cooled to 10°C. Recovery required the greatest number of twitches after continuous optical stimulation. (a) At 1, 10, 25, and 50 Hz and continuous light, twitch force amplitudes recovered after 1 ± 1, 4 ± 3, 8 ± 4, 8 ± 3, and 16 ± 6 twitches to within 2 standard deviations of the average peak force of the final 10 twitches (n = 6 mice, 90 trials). Error bars represent mean ± SEM calculated from the within mouse variances of mean. (b) Example traces from one mouse demonstrate recovery at increasing frequencies of optical stimulation where force is normalized to the average pre-light amplitude.

We quantified the inhibition of twitch force amplitudes with 1, 10, 25, and 50 Hz blue light pulses and continuous light in *Thy1-ChR2-EYFP* and wild-type mice while the nerve was maintained at approximately 10°C (Fig. 2). The average twitch force amplitude during optical stimulation as a percentage of the average pre-light force amplitude decreased significantly with increasing light pulse frequency (p = 2 × 10^−13^) in *Thy1-ChR2-EYFP* mice. Twitch force amplitudes during optical stimulation were unchanged in wild-type mice, and there was no significant effect of light pulse frequency in wild-type mice (p = 0.856). We observed slower recovery of post-light twitch force amplitudes after inhibition using higher light pulse frequencies (p = 7.8 × 10^−6^, Fig. 3).

### Effect of nerve temperature on the response to optical stimulation

**Figure 4:**
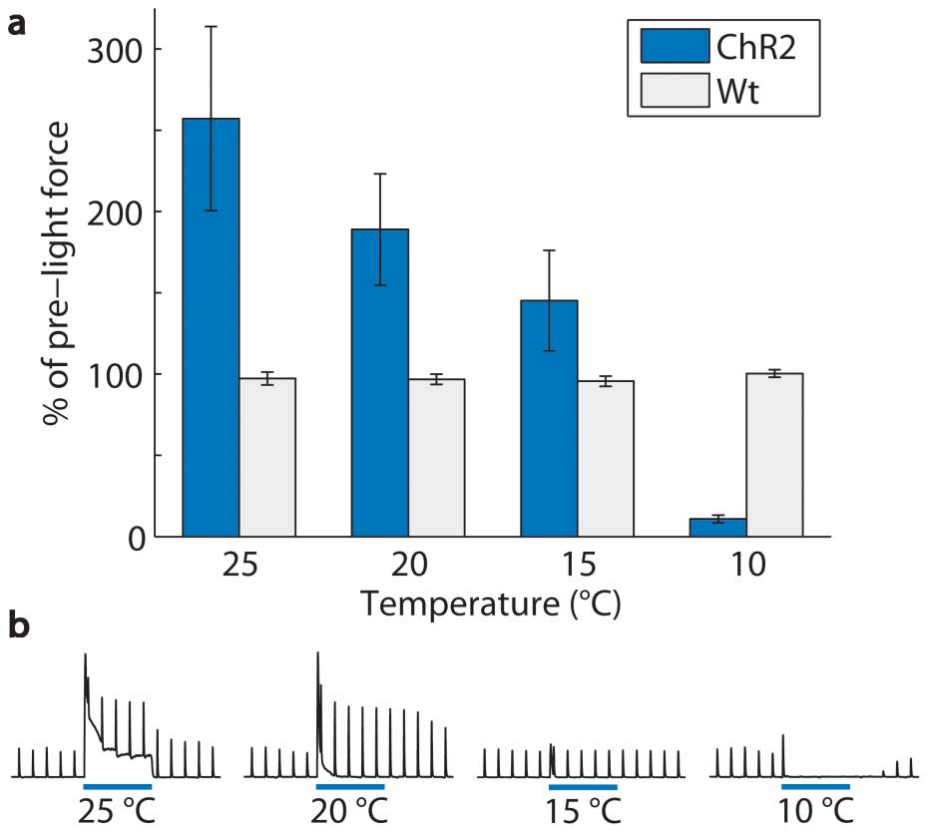
Muscle activity in response to optical stimulation (continuous light, 5 s pulse duration, 7 mW/mm^2^) in *Thy1-ChR2-EYFP* mice (n = 6 mice, 72 trials) transitioned from enhancement to inhibition with decreasing temperature of the nerve. (a) At 25, 20, and 15°C, average twitch force amplitudes increased to 257 ± 57, 189 ± 34, and 145 ± 31% of the pre-light amplitudes. At 10°C, average twitch force amplitude was inhibited to 11 ± 2% of the pre-light amplitude. Average twitch force amplitudes in wild-type mice (Wt, n = 4 mice, 48 trials) were unchanged during optical stimulation (97 ± 4, 97 ± 3, 96 ± 3, and 100 ± 2% at 25, 20, 15, and 10°C). Pre-light twitch force amplitudes were approximately 50% of maximal in all trials. Error bars represent mean ± SEM calculated from the within mouse variances of mean. (b) Example traces from one mouse show average twitch force amplitudes of 284, 262, 99, and 1% of pre-light amplitudes at 25, 20, 15, and 10°C.

We examined the role of nerve temperature in achieving optical inhibition using continuous blue light (5 s pulse duration, 7 mW/mm^2^) with the nerve maintained at 25, 20, 15, and 10°C. At warmer temperatures of 25, 20, and 15°C, optical stimulation increased twitch force amplitudes (Fig. 4). This enhancement of electrically evoked twitch force amplitudes indicates that optical stimulation of ChR2 at the distal nerve was able to enhance the electrical recruitment of motor axons at the proximal nerve. Further cooling of the nerve, however, produced a transition from enhancement to inhibition. Cooling the nerve below 15°C was important for achieving reliable optical inhibition in *Thy1-ChR2-EYFP* mice. Under these conditions, optical stimulation of ChR2 at the distal nerve inhibited transmission of electrically evoked action potentials and thus inhibited twitch forces. The average twitch force amplitude during optical stimulation as a percentage of the average pre-light force amplitude decreased significantly with decreasing nerve temperature (p = 3.63 × 10^−12^). Twitch force amplitudes in wild-type mice (n = 4 mice, 48 trials) were unchanged during optical stimulation, and there was no significant effect of temperature in wild-type mice (p = 0.174). Effects of optical stimulation or electrical stimulation at each temperature are shown in Supplementary Table S1.

**Figure 5:**
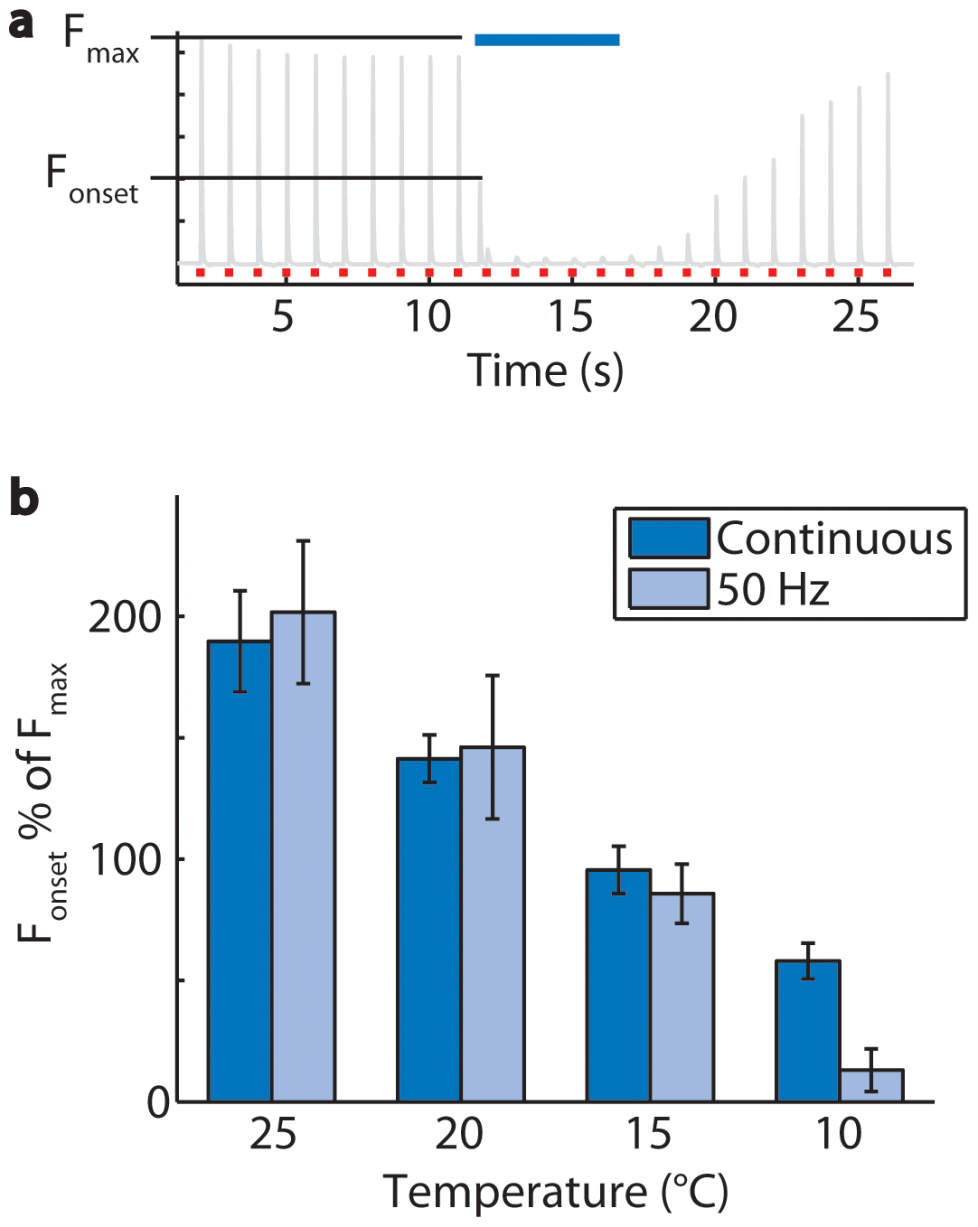
Onset forces caused by optical stimulation in *Thy1-ChR2-EYFP* mice. (a) The peak force caused by the onset of optical stimulation (F_onset_) is analyzed as a percent of the maximal twitch force for each mouse (F_max_). (b) In trials performed at 25, 20, 15, and 10°C, the onset forces caused by continuous optical stimulation were 190 ± 21, 141 ± 10, 96 ± 10, and 58 ± 7% of the maximal twitch force for each mouse (n = 6 mice, 18 trials per frequency). Onset forces caused by 50 Hz optical stimulation were 202 ± 29, 146 ± 30, 86 ± 12, 13 ± 9% of the maximal twitch force for each mouse. Error bars represent mean ± SEM calculated from the within mouse variances of mean.

The onset of optical stimulation caused a peak in force in some trials (Fig. 5a). This onset force was nearly twice the maximal twitch force amplitude at a nerve temperature of 25°C and decreased significantly with decreasing temperature of the nerve (p = 9.82 × 10^−12^, Fig. 5b). At 10°C, the temperature favorable for inhibition, the onset force was less than the maximal twitch force. Onset forces caused by 50 Hz light pulses and continuous light were similar.

### Simultaneous all-optical excitation and inhibition using a single opsin and single light wavelength

**Figure 6:**
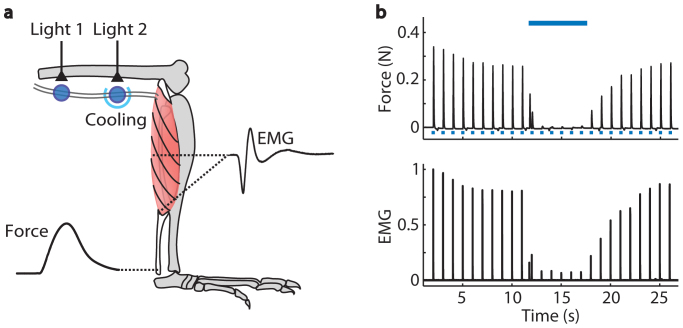
Simultaneous optical excitation and optical inhibition of motor neuron and muscle activity in a *Thy1-ChR2-EYFP* mouse. (a) Schematic of experimental measures during simultaneous optical excitation and optical inhibition of motor neuron and muscle activity in a *Thy1-ChR2-EYFP* mouse. Motor axons were optically stimulated with blue laser light (465 nm, 7 mW/mm^2^) at the proximal sciatic nerve. The distal nerve was cooled and illuminated with blue laser light from a second laser (473 nm, 7 mW/mm^2^). EMG and force were recorded from the lower limb muscles. (b) Example force and EMG data recorded during muscle twitches elicited by 25 s of 1 Hz blue light pulses (2 ms pulse duration, 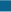, n = 1 mouse, 1 trial). EMG was full-wave rectified, filtered, and normalized to the maximum amplitude recorded during the trial. Average force and EMG were inhibited to 0% and 3%, respectively, of the pre-light amplitudes during 5 s of continuous light (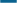) with the nerve cooled to 7°C.

We discovered that it is possible to simultaneously control both excitation and inhibition of motor neuron activity with blue light in *Thy1-ChR2-EYFP* mice (Fig. 6). Motor neuron activity was evoked with 1 Hz (2 ms pulse duration, 7 mW/mm^2^) light pulses directed at the proximal nerve, and that activity was inhibited with a continuous 5 s light pulse (7 mW/mm^2^) directed at the distal nerve from a second laser light source. Simultaneous optical excitation and inhibition by blue light stimulation of ChR2 is also demonstrated in Supplementary Video S1 and Supplementary Figure S1.

## Discussion

Our results demonstrate that ChR2 can both enhance and block transmission of electrically evoked action potentials. We propose that ChR2 photocycle kinetics underlie this bifunctionality. Sufficient illumination opens ChR2 channels, enabling cation influx and membrane depolarization that can evoke action potential firing^22^. The time constants of ChR2 opening and closing, 10 and 11 ms respectively^34^,^35^, are slow in comparison with native ion channels^36^ and limit the ability of ChR2 to evoke trains of action potentials upon optical stimulation with high frequency light pulses^22^,^37^,^38^,^39^ or continuous^40^ light. For example, increasing light pulse frequency from 10 to 50 Hz can cause a 36% reduction in ChR2 channel closing^40^ and prevents complete membrane repolarization by native ion channels and subsequent action potential firing. Instead, increasing light pulse frequency results in summation of the membrane potential, referred to as the plateau potential^38^,^40^,^41^ and observed to exceed 15 mV sustained depolarization during optical stimulation^38^,^40^. Notably, this plateau potential is not observed in experiments using an engineered ChR2 variant with faster photocycle kinetics^38^. Further, ChR2 photocycle kinetics are temperature and voltage sensitive such that they are slowed by cooling or membrane depolarization, and cooling results in a more depolarized plateau potential^41^. We suggest that optically evoked action potentials and sustained membrane depolarization are critical to understanding why ChR2 can both enhance and block transmission of electrically evoked action potentials.

To explain why ChR2 can enhance electrically evoked action potentials, we reasoned that optically evoked action potentials transmit orthodromically and antidromically from the site of illumination. Orthodromic action potentials generate muscle force, which we observed during continuous light as tetanic force at 25°C and as onset forces at all temperatures (Figure 4). We suggest that the corresponding antidromic action potentials may have depolarized the average baseline membrane potentials of axons at the site of electrical stimulation proximal to the site of illumination. This would raise the probability that electrical stimulation would evoke action potentials and therefore enhance motor unit recruitment and twitch force amplitudes, which we observed with the nerve maintained at 25°C (Figure 4). We also observed enhancement of twitch force amplitudes with the nerve maintained at 20 and 15°C. However, at these lower temperatures we observed minimal optically evoked force during the 5 s continuous light, suggesting that there were few optically evoked action potentials. Taken together these results suggest that antidromic optically evoked action potentials may not be necessary but that subthreshold optically evoked membrane depolarization may be sufficient to depolarize the baseline membrane potential at the site of electrical stimulation and enhance electrically evoked twitch force amplitudes.

To explain why ChR2 can inhibit transmission of electrically evoked action potentials, we considered that cooling reduces the ability of ChR2 to evoke trains of action potentials and results in greater depolarization of the plateau potential^41^. With the nerve maintained at 10°C we observed no optically evoked force after the onset force, and we suggest that there was greater sustained membrane depolarization at the site of illumination. Sustained membrane depolarization holds native voltage-dependent sodium channels in the inactive state, thereby preventing sodium channels from opening to initiate or transmit action potentials^36^. This sodium channel inactivation is a commonly-proposed mechanism of inhibition by high frequency^6^,^8^,^9^,^14^,^42^ or continuous^4^,^7^,^10^,^11^,^17^ electrical stimulation. We suggest that at 10°C the membrane was sufficiently depolarized to inactivate sodium channels and inhibit action potential transmission through the site of illumination. While native sodium channels are central to this mechanism, other native membrane components may have contributed. For example, other transmembrane channels^43^,^44^ and cellular receptors^45^ display functional changes upon ChR2 evoked membrane depolarization.

We suggest that the role of decreasing temperature in the response to optical stimulation was to slow ChR2 and native channel dynamics until the sustained membrane depolarization was sufficient for inhibition. We suggest that the role of increasing light pulse frequency in achieving optical inhibition at 10°C was to decrease the time between optically evoked depolarizations and thus reduce the ability of native channels to adequately repolarize the membrane potential.

Optical inhibition was greatest above a threshold frequency of 50 Hz. High frequency electrical inhibition also has a minimum frequency above which inhibition is achieved^12^,^13^,^15^,^21^ which ranges from 200 Hz to 30 kHz^1^. Cooling reduces the frequency threshold for electrical inhibition both in computer simulations^13^,^16^,^21^ and *in vivo*^12^. We propose that slow ChR2 photocycle kinetics in comparison with native channel kinetics in combination with cooling of the nerve may explain the lower frequency threshold observed for optical inhibition relative to thresholds for high frequency electrical inhibition of mammalian peripheral neurons.

Onset forces caused by optical inhibition were reduced by cooling the nerve and were less than the maximal twitch force amplitude when the nerve temperature was 10°C. A previous investigation of electrical inhibition of sciatic motor neurons in rats reports onset forces of one to eight times the maximal single twitch force amplitude, with the onset forces varying from a single twitch to a summed twitch response^5^,^6^. These large onset forces caused by electrical inhibition present a significant obstacle for clinical implementation.

The all-optical system we present here is free from stimulation-induced electrical artifacts. In contrast, investigations into the mechanisms of electrical inhibition have been limited by electrical artifacts that interfere with electrophysiological recording of neuronal membrane potentials^2^,^12^,^21^. To date, computer simulations employing mammalian axon models are the primary tool available to study mechanisms of high frequency inhibition^11^,^21^. Optical inhibition provides an entirely new tool that when combined with *in vivo* or *in vitro* electrophysiology has potential to reveal long sought insights into the mechanisms of high frequency inhibition. Experimental evidence of the mechanisms of high frequency inhibition will inform further studies to optimize stimulation parameters and pursue clinical applications^8^,^9^,^13^,^15^,^21^. The ability to inhibit neuronal activity using ChR2 may circumvent challenges of NpHR expression and thus broaden the *in vivo* and *in vitro* systems available to study optogenetic inhibition and combined excitatory and inhibitory optical control.

## Methods

### *In vivo* measurements

All animal procedures were approved by the Stanford University Institutional Animal Care and Use Committee in adherence with the National Institutes of Health Guide for the Care and Use of Laboratory Animals. Healthy adult male and female *Thy1-ChR2-EYFP* and wild-type C57BL/6 mice of average mass 34 g and average age 23 weeks were studied. *Thy1-ChR2-EYFP* (line 18) mice are available from The Jackson Laboratory (Bar Harbor ME). We performed a series of experiments to determine if electrically and optically evoked motor neuron and muscle activity could be inhibited by illumination of the sciatic nerve in anesthetized *Thy1-ChR2-EYFP* and wild-type mice (Supplementary Fig. S2). Mice were anesthetized with isoflurane (1–3%) and kept warm on an electrical heat pad (37 °C). The hindlimb was shaved, and the sciatic nerve was exposed. The triceps surae muscle group of the lower limb was exposed, and the Achilles tendon was isolated. The calcaneus was cut, and a small bone piece at the tendon was attached through a lightweight, rigid hook to a force transducer (0.3 mN resolution, 300C-LR, Aurora Scientific, Aurora, Ontario). EMG electrodes made from stainless steel, tetrafluoroethylene-coated wires (50 μm, 790700, A-M Systems, Carlsborg WA) were inserted into the medial gastrocnemius muscle belly and muscle-tendon junction with a ground electrode at the forelimb wrist.

To electrically evoke motor neuron activity we placed a custom built stimulation cuff around the sciatic nerve and applied electrical stimulation. The sciatic nerve includes motor and sensory axons; however, only motor activity was investigated here. Electrically stimulated motor axons propagate action potentials to the lower limb muscles to evoke contractile and electrical muscle activity. Contractile activity was recorded by the force transducer, and electrical activity was recorded by the EMG electrodes. To achieve optical inhibition, we illuminated axons in the nerve approximately 1 cm distal to the electrical stimulation cuff with blue laser light.

### Electrical stimulation

Motor axons were electrically stimulated (S48 Stimulator, Grass Technologies, West Warwick RI) through the stimulation cuff. Maximal force amplitude was determined for each mouse at the start of the experiment by incrementally increasing voltage until twitch force amplitudes plateaued. Experiments were then performed using a stimulation voltage that evoked either 100% (Fig. 1–3) or 50% (Fig. 4–5) of maximal twitch force amplitudes in each mouse. Experiments performed at 100% of maximal electrical stimulation and 10°C, a nerve temperature at which we observed reliable inhibition, recruited approximately all motor units such that we could investigate optical inhibition of the entire motor neuron pool. Experiments performed at 50% of maximal electrical stimulation recruited only a portion of motor units such that we could determine if the addition of optical stimulation caused enhancement or inhibition of electrically evoked twitch forces at each temperature.

### Illumination

Illumination at the distal nerve was from a blue laser (465 nm, OEM Laser Systems, East Lansing MI) via a multimode optical fiber (365 μm diameter, 0.37 numerical aperture, Thorlabs, Newton NJ) (Supplementary Fig. S2a, b). The 465 nm wavelength is within 2% of the ChR2 activation peak of 470 nm^23^. Light power density was calculated as the power at the fiber tip measured with a power meter (PM100, Thorlabs) divided by the light spot area of 1 mm^2^ illuminating the nerve (light spot diameter of approximately 1.1 mm). The internodal length of C57BL/6 mouse sciatic nerve is approximately 0.63 mm^46^. Therefore, we expect that this light spot illuminated on average 1–2 nodes of Ranvier per axon. The angle of light incidence at the nerve surface was insufficient to cause total internal reflection assuming the nerve refractive index is similar to that of brain^47^. We therefore expect that light propagated transversely through the nerve with minimal longitudinal spreading. Experiments with *Thy1-ChR2-EYFP* and wild-type mice involved 2 to 4 hours of repeated electrical and optical stimulation (up to 150 trials per mouse). We observed no localized nerve damage and no systematic change in force or EMG during this time.

Each trial of 25 twitches included consecutively 10 pre-light, 5 with light, and 10 post-light twitches. Each trial of 65 twitches included consecutively 10 pre-light, 5 with light, and 50 post-light twitches. The start, end, frequency, and pulse duration of the light within each trial was controlled using MATLAB (Mathworks, Natick MA) and interfacing with a waveform generator (33220A, Agilent Technologies, Santa Clara CA) to trigger light pulses from the laser. Figure 4 includes tests of 1, 10, 25, and 50 Hz with 2 ms pulse widths and continuous light for 5 seconds. The order of trials of each combination of frequency and pulse duration was randomized within experiments to avoid temporal effects.

We used two lasers for experiments using both optical excitation and optical inhibition (Supplementary Fig. S2c–f). A multimode optical fiber from a second laser (473 nm, OEM Laser Systems) was positioned at the proximal nerve to excite twitch forces. The 473 nm wavelength is within 1% of the ChR2 activation peak of 470 nm^23^. We refer to using a single wavelength of light (approximately 470 nm). This optical fiber replaced the electrical stimulation cuff used for experiments represented in Figures 1–5. Illumination at the distal nerve was from an optical fiber positioned approximately 1 cm distal on the nerve to inhibit motor neuron activity. Sufficient distance between the two light spots along the nerve is critical. Overlapping light spots on the nerve would prevent the distinct excitation of motor axons by one light source and inhibition by a second light source.

### Nerve cooling

Nerve temperature was controlled with a custom built nerve cooling cuff incorporating a miniature Peltier thermode (00301-9X39-10RU2, Custom Thermoelectric, Bishopville MD) and a heat sink (Supplementary Fig. S3). Voltage input to the Peltier thermode was adjusted to control nerve temperature within the range studied. Data represented in Figure 2 were collected at 10 ± 2 °C for *Thy1-ChR2-EYFP* mice and 11 ± 1 °C for wild-type mice. Data represented in Figure 3 were collected at nerve temperatures of approximately 25, 20, 15, and 10 °C. Actual mean ± SEM temperatures used for trials represented in Figure 4 were 25 ± 1, 19 ± 2, 15 ± 1, and 9 ± 3 °C for *Thy1-ChR2-EYFP* mice and 26 ± 0, 21 ± 1, 16 ± 1, and 11 ± 1 °C for wild-type mice. All trials within each mouse were performed with the nerve temperature maintained at approximately 25 °C, followed by 20, 15, and 10 °C to avoid repeated cooling and warming of the nerve.

Nerve temperature was approximated with a thermocouple (K-type, 36 gauge, Custom Thermoelectric) in contact with the cooling cuff. Temperature data were recorded using a National Instruments CompactDAQ Chassis (cDAQ-9171, National Instruments, Austin TX) with thermocouple input module (NI 9211, National Instruments).

### Data recording and analysis

Force and EMG data were recorded at 10 kHz (PCI-6251, National Instruments) and analyzed using MATLAB. Passive force amplitude (muscle force with no activation) was measured for each trial prior to electrical stimulation of the nerve. Analyses were done on the active (total – passive) force. Average peak twitch force pre-light (F_av_pre-light_) and during optical stimulation (F_av_light_) were calculated for each trial of 25 twitches. We calculated the percent to which average force was inhibited by light. 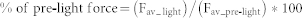
EMG was bandpass filtered (3–3000 Hz) and full-wave rectified, and percent of pre-light EMG was calculated using the same method as was used for force. EMG amplitudes were normalized to the maximum EMG amplitude observed in that trial.

We quantified the number of post-light twitches required for force amplitude to recover after inhibition. We defined recovery as the first post-light twitch having a peak force within 2 standard deviations of the average peak force of the final ten twitches of the trial.

### Histology

**Figure 7:**
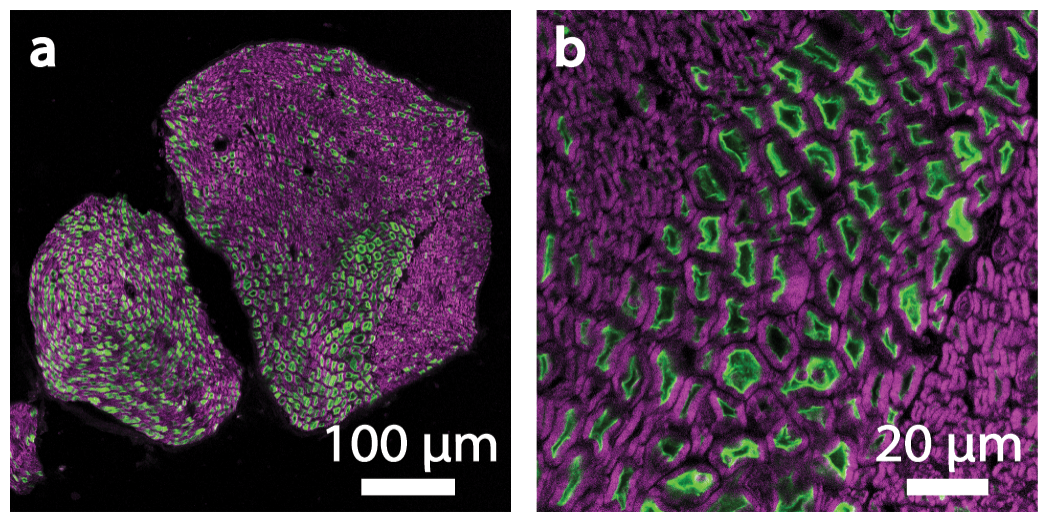
ChR2 expression in the sciatic nerve of a *Thy1-ChR2-EYFP* mouse. (a) Confocal image of a *Thy1-ChR2-EYFP* mouse sciatic nerve in cross section distal to bifurcation showing motor neurons expressing ChR2-EYFP (green). A fluorescent myelin stain (magenta) labels myelin surrounding neurons. (b) Magnified image.

*Thy1-ChR2-EYFP* mice express ChR2 fused to enhanced yellow fluorescent protein (EYFP) in the peripheral nervous system. We performed histology of 20 μm thick cryosections of sciatic nerve to confirm ChR2 expression in motor neurons of *Thy1-ChR2-EYFP* mice (Fig. 7). We washed sections in PBS and permeabilized sections in PBS and 0.2% Triton X-100 for 20 min. We treated sections with a fluorescent myelin stain (1:300, FluoroMyelin, Invitrogen, Grand Island NY), rinsed in PBS, and mounted with PVA-DABCO (Sigma-Aldrich, Saint Louis MO) prior to confocal microscopy. Scans in the EYFP channel were taken at 40× and 63× magnifications at a gain and offset such that no fibers were saturated. Image brightness and contrast were adjusted using ImageJ^48^. ChR2 expression was sufficiently high that all axons expressing ChR2 in nerves from transgenic mice were brighter than the background levels observed in axons from wild-type mice.

### Statistics

Standard errors of the mean (SEM) were calculated as the squared sums of the within-mouse variances of mean calculated for each mouse. P values representing the effects of frequency and temperature were calculated using simple linear regression models (α < 0.01). Frequencies of 1, 10, 25, and 50 Hz were included in the linear regression model, while continuous light was not included.

## Author Contributions

H.L. designed and conducted the experiments and performed analyses. X.Q., P.A., K.D. and S.D. advised the experiments. H.L., X.Q., P.A. and S.D. wrote the manuscript.

## Supplementary Material

Supplementary Information Supplementary Information

Supplementary Information Supplementary Video S1
