# Supplementary material for: Optical control of neuronal excitation and inhibition using a single opsin protein, ChR2
Source: Sci Rep. 2013 Oct 31;3:3110. doi: 10.1038/srep03110 (PMC3813941; doi:10.1038/srep03110)
Supplement: Supplementary Information [file srep03110-s1.pdf]

Supplementary Information

Optical control of neuronal excitation and inhibition using a single opsin protein, ChR2

Holly Liske[1], Xiang Qian[2], Polina Anikeeva[3], Karl Deisseroth[4,5], Scott Delp[1,5,*]

Departments of [1]Mechanical Engineering, [2]Anesthesiology, Perioperative, and Pain Medicine, [4]Psychiatry and Behavioral Sciences, and [5]Bioengineering, Stanford University, Stanford, California 94305, and Department of [3]Materials Science and Engineering, Massachusetts Institute of Technology, Cambridge, Massachusetts 02139. [*]Correspondence and requests for materials should be addressed to S.D. (email: delp@stanford.edu).

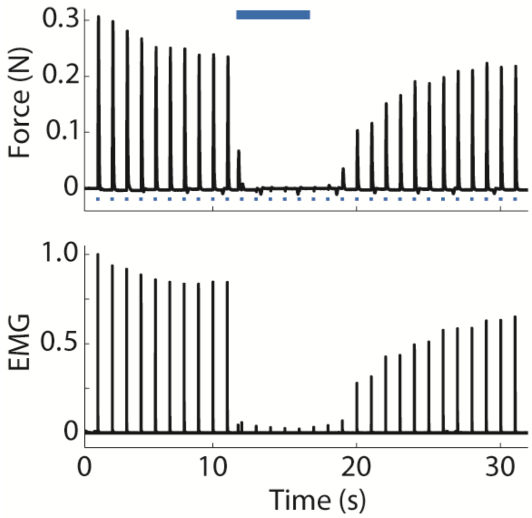

Supplementary Figure S1. Example force and EMG data recorded during the trial shown in Supplementary Video S1 (n = 1 mouse). EMG was full-wave rectified, filtered, and normalized to the maximum amplitude recorded during the trial. Force and EMG were evoked by 30 s of 1 Hz blue light pulses (1 ms pulse duration, ■, n = 1 mouse, 1 trial) and inhibited to 1% and 3%, respectively, by 5 s of continuous light (7 mW/mm^2, ▬) with the nerve cooled to 9 °C.

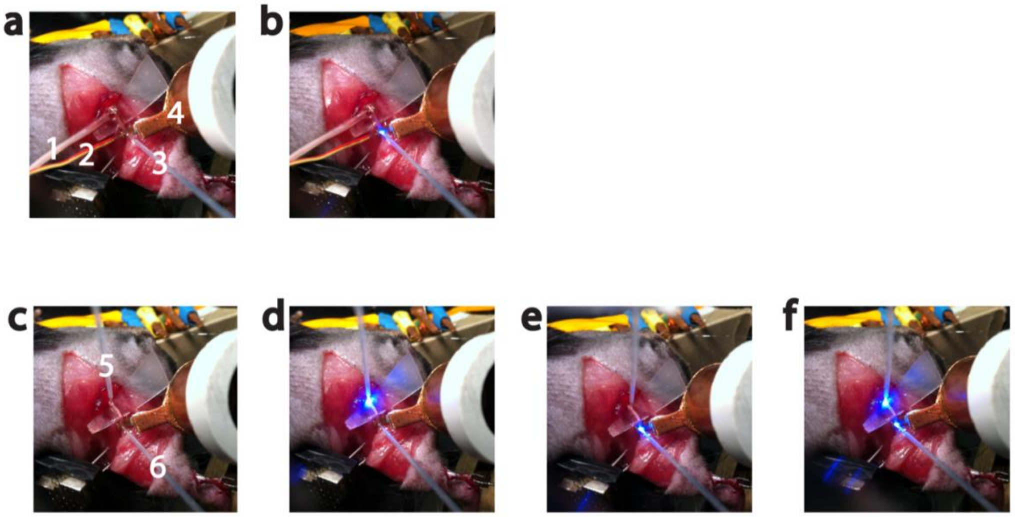

Supplementary Figure S2. *In vivo* animal preparation for electrical excitation and optical inhibition (**a-b**) and for optical excitation and optical inhibition (**c-f**). (**a**) Electrical stimulation cuff at proximal nerve (**1**), thermocouple (**2**), optical fiber at distal nerve (**3**), and cooling cuff (**4**). (**b**) Preparation in (**a**) during optical inhibition with blue light. (**c**) Optical fiber at proximal nerve (**5**) to excite and optical fiber at distal nerve (**6**) to inhibit motor neuron and muscle activity. (**d**, **e**, **f**) Preparation in (**c**) during optical excitation (**d**), optical inhibition (**e**), and simultaneous optical excitation and inhibition (**f**).

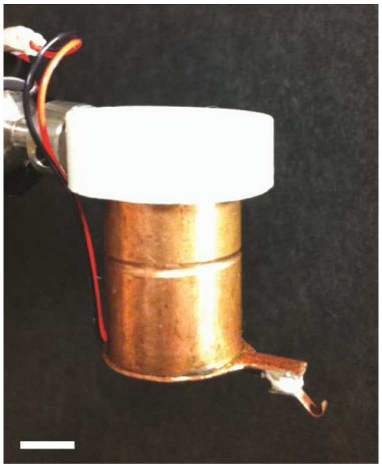

Supplementary Figure S3. Cooling cuff incorporating a miniature Peltier thermode to control the temperature of the nerve. Scale bar, 5 mm.

Supplementary Table S1. Example force responses to optical stimulation of the distal nerve at each frequency and temperature (columns **a**), electrical stimulation of the proximal nerve at 1 Hz and each temperature (column **b**), and optical stimulation of the proximal nerve at 1 Hz and each temperature (column **c**, n = 1 mouse). Force amplitudes in response to optical stimulation of ChR2 at the location of cooling on the distal nerve decreased with decreasing temperature, as seen by comparing force amplitudes between the rows of columns **a**; although, this trend was not consistent for 1 Hz optical stimulation. However, force amplitudes in response to electrical or optical stimulation at the proximal nerve did not change with decreasing temperature at the location of cooling on the distal nerve, as seen by the similar force amplitudes in the rows of columns **b** and **c**, respectively. While others have demonstrated a cold block of nerve conduction[1], this further demonstrates that cooling of the nerve to the temperatures studied here was not sufficient to cause inhibition.

<table>
<tr><td rowspan="2">Nerve temperature (°C)</td><td colspan="5">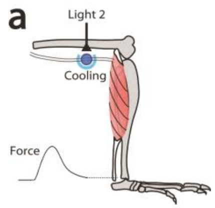</td><td>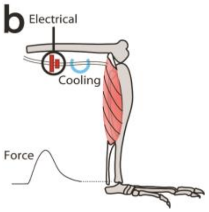</td><td>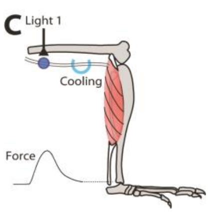</td></tr>
<tr><td>Continuous</td><td>50 Hz</td><td>25 Hz</td><td>10 Hz</td><td>1 Hz</td><td>1 Hz</td><td>1 Hz</td></tr>
<tr><td>25</td><td>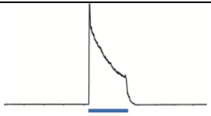</td><td>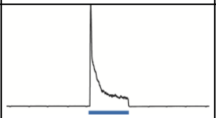</td><td>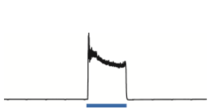</td><td>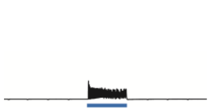</td><td>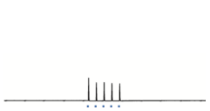</td><td>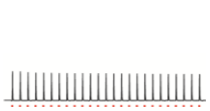</td><td>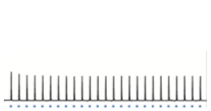</td></tr>
<tr><td>20</td><td>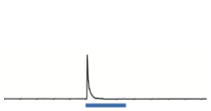</td><td>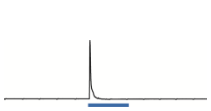</td><td>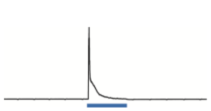</td><td>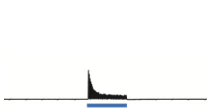</td><td>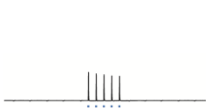</td><td>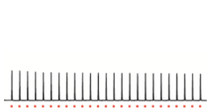</td><td>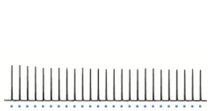</td></tr>
<tr><td>15</td><td>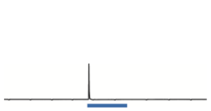</td><td>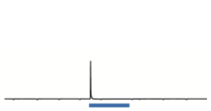</td><td>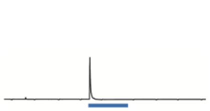</td><td>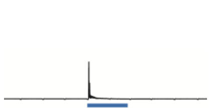</td><td>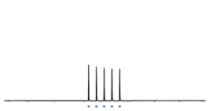</td><td>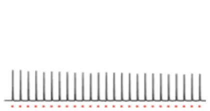</td><td>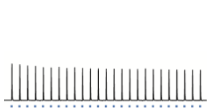</td></tr>
<tr><td>10</td><td>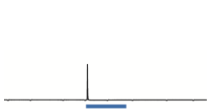</td><td>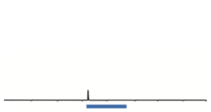</td><td>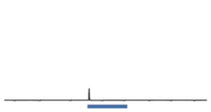</td><td>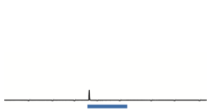</td><td>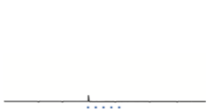</td><td>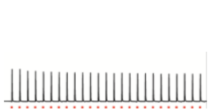</td><td>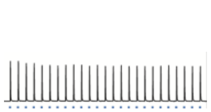</td></tr>
</table>

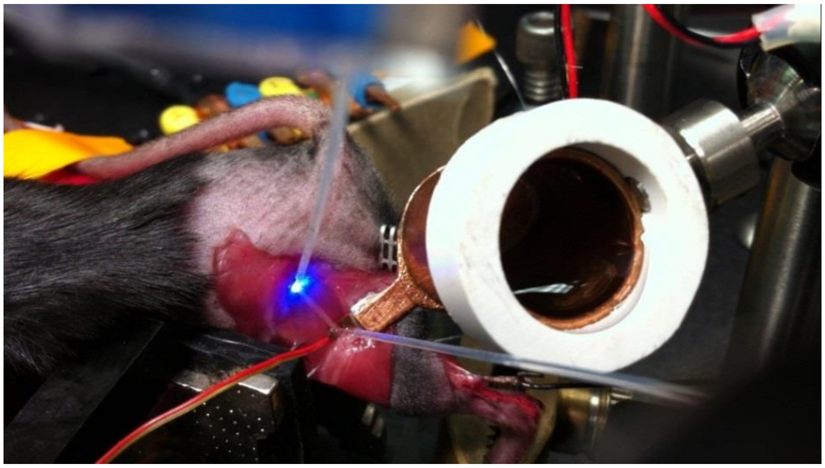

Supplementary Video S1. Simultaneous optical excitation and optical inhibition of motor neuron and muscle activity in a *Thy1-ChR2-EYFP* mouse. An optical fiber positioned at the proximal sciatic nerve evoked motor neuron and muscle activity with 30 s of 1 Hz pulses of blue light (1 ms pulse duration, 5 mW/mm^2). A second optical fiber positioned at the distal nerve inhibited the activity with a 5 s continuous blue light pulse (7 mW/mm^2) while the nerve was cooled to 9 °C. Force and EMG data are presented in Supplementary Figure S1.

References

1. Paintal, A.S. Block of conduction in mammalian myelinated nerve fibres by low temperatures. *J Physiol* **180**, 1-19 (1965).
